# Effects of short-term in-season break detraining on repeated-sprint ability and intermittent endurance according to initial performance of soccer player

**DOI:** 10.1371/journal.pone.0201111

**Published:** 2018-08-15

**Authors:** Alejandro Rodríguez-Fernández, Javier Sánchez-Sánchez, Rodrigo Ramirez-Campillo, José Antonio Rodríguez-Marroyo, José Gerardo Villa Vicente, Fabio Yuzo Nakamura

**Affiliations:** 1 Institute of Biomedicine (IBIOMED), Department of Physical Education and Sports, University of León, León, Spain; 2 Faculty of Physical Activity Sciences and Sports, University Isabel I, Burgos, Spain; 3 Unit Assessment and Planning of Sports Training, Faculty of Education, Pontifical University of Salamanca, Salamanca, Spain; 4 Department of Physical Activity Sciences, Universidad de Los Lagos, Osorno, Chile; 5 Department of Medicine and Aging Sciences “G. d´Annunzio” University of Chieti-Pescara, Chieti, Italy; 6 The College of Healthcare Sciences, James Cook University, Townsville, Queensland, Australia; National Center of Medicine and Science in Sport, TUNISIA

## Abstract

To better understand the detraining effects in soccer, the purpose of the study was to analyse if performance level of soccer players modulate repeated-sprint ability (RSA) and intermittent endurance changes during 2-weeks of detraining (i.e., in-season break). Seventeen professional and sixteen young elite soccer players of two different teams performed, before and after 2-weeks of detraining, the RSA test and the Yo-Yo Intermittent Recovery Test, level 1 (YYIR1). Before detraining, professional players perform better (p < 0.05) RSA best time (RSA_best_) than young players. A decrease (p < 0.05) in RSA_best_, RSA total time (RSA_total_) and mean time (RSA_mean_) performance was observed in both teams, without changes in RSA fatigue index (*Sdec)*. No significant changes in distance covered during YYIR1 was observed in any team. Before detraining, faster players from both teams (FG) (following the median split technique, soccer players with RSA_best_ ≤ 3.95 s) performed better (p < 0.01) in RSA_total_, RSA_mean_ and RSA_best_, but worse (p < 0.01) in *Sdec*. Although FG and the slower players (SG, RSA_best_ > 3.95 s) showed a worse (p < 0.05) RSA_total_, RSA_best_ and RSA_mean_ performance after detraining (ES = 1.5, 1.4 and 2.9; ES = 0.6, 1.2 and 0.6; for FG and SG, respectively), the deterioration was greater in the FG for RSA_best_ (p < 0.05) and RSA_total_ (ES = 1.46). After detraining, FG improved (p < 0.05) *Sdec* performance. In conclusion, a 2-week in-season break (detraining) period induced a worse RSA, with no effect on intermittent endurance in professional and elite young soccer players, with greater detrimental effects on RSA_total_ and RSA_best_ in FG. In addition, *Sdec* does not seem to be sensitive to changes in RSA after a 2-week in-season break.

## Introduction

The ability to perform short-duration multiple sprints interspersed with short recovery times has been termed “repeated-sprint ability” (RSA) [1]. Although debate exists regarding the main factors determining soccer performance [2], the importance of RSA is recognized [3,4]. For instance, significant correlation exists between very-high intensity running distances covered during matches and mean sprint time on a RSA test [5]. Besides this, single and repeated-sprint efforts are frequently involved in crucial moments of match-play [6], including creation of goal scoring opportunities. Therefore, constant evaluation of RSA throughout the season can provide valuable information to coaches and athletes.

In addition to RSA, intermittent high-intensity endurance is also considered crucial to performance in soccer [7]. Although the importance of total running distance covered at high-intensity in soccer could be masked by the technical-tactical level of a team [8], players at a higher standard of competition tend to perform significantly more high-intensity running than those at a lower standard [9], and this ability can be assessed by the Yo-Yo intermittent recovery test, level 1 (YYIR1) [10]. Previous studies have shown no effects of detraining after one week in the Yo-Yo Intermittent Recovery Test, level 2 [11]. However, significant (p < 0.01) detrimental effects of ~5 and ~22% after three days and 2-weeks of inactivity, respectively, was reported in a study [12]. Aside from the lack of agreement across studies, to our knowledge, no study has analyzed the effects of short-term detraining after an in-season break using the YYIR1 as a marker of performance. Despite YYIR1 and YYIR2 test performances correlate very largely [13], the lower level of speed effort required by the YYIR1 might better detect changes in aerobic fitness of players after a detraining period.

The effects of different training programs on RSA have been assessed in soccer players [14–17]. However, the impact of in-season unloading or detraining on RSA in soccer players remains unresolved. For instance, previous studies have analyzed the effects of 1- [11] or 2-week [12,18] off-season detraining periods on RSA (complete interruption of training), showing detrimental effects of ~2% after two weeks for RSA total time and ~3% for sprinting speed in the last five repetitions during a 20-m RSA test with a total of 10 repetitions [18]. However, RSA best time and RSA fatigue index were not affected after 2-weeks of inactivity [12,18]. Possibly, the loading pattern (e.g., deliberate overload leading to overreaching) and the athlete’s performance level previous to detraining might modulate such changes. A better understanding of this phenomenon is relevant since most soccer leagues encompass a period without competitive matches [19] or in-season break detraining period.

Similar to off-season breaks, in-season breaks can also lead to short-term detraining [12,18,20]. This may induce cardiovascular and neuromuscular deconditioning [21], which can potentially impair RSA. Notably, RSA is related to chronological age [1], competitive level [4] and intermittent high-intensity endurance [22]. However, the interaction between detraining, RSA, age, competitive level, and intermittent high-intensity endurance is unknown and needs to be clarified.

RSA is usually assessed by the total time (RSA_total_) [23], mean time (RSA_mean_) [4,24], best time (RSA_best_) [14], and fatigue index or the percentage decrement score (*Sdec*) [25]. Although *Sdec* is considered a reliable RSA marker [26], recent research has suggested that “absolute” performance values (i.e., total, mean and best times) can be more reliable and sensitive to training effects [4,27]. In response to detraining *Sdec* showed contradictory results, with significant (p = 0.04) detrimental changes (5.8 ± 2.8% to 7.8 ± 3.2%) [11] or no significant changes (5.9 ± 2.3% to 7.6 ± 2.8%) [12] after 1 or 2-week of detraining, respectively. The knowledge of the extent of RSA responses to a short-term detraining period can help physical trainers to foresee eventual changes during the in-season breaks, and to implement adequate training strategies to optimize fitness levels and training time after returning from the break.

Therefore, the main aim of the study was to analyse if initial performance level (sprinting speed) of soccer players modulates repeated-sprint ability (RSA) and intermittent endurance changes during 2-weeks of detraining (i.e., in-season break). A substantial detraining effect for both professional senior and young elite soccer players and the influence of “baseline” fitness level (pre-detraining) on this effect were assumed as the working hypotheses of this study.

## Material and methods

### Study design

The 2-week in-season break period took place during the mid-phase of the season (i.e., Christmas holidays), with players not involved in any competitive game or training session (team or individual session) during this period. Specifically, players were asked to refrain for any type of physical activity training (other than daily life physical activity) during the in-season break. After the break, players were individually interviewed to assess the level of accomplishment of the aforementioned requirement. In addition, players were asked to reduce meaningful changes in their diet, although this was not controlled. The same training week was reproduced before and after the 2-week Christmas break (Table 1). The tests were imbedded into the training sessions, so that there was no disturbance to the training plan. During day one, anthropometry (ISAK procedure) and RSA test were performed (16:30 to 18:30 pm) and, during day two, the YYIR1 was completed (16:30 pm) and used to estimate VO_2_max of soccer players [13]. Prior to the two exercise tests, soccer players performed the same warm-up (i.e., low-intensity running, dynamic flexibility, 20-m run-ups). Participants were fully familiarized with testing protocols, which were routinely performed in the respective investigated clubs. Athletes refrained from vigorous high-intensity exercise 24 hours before testing sessions and were instructed to maintain normal daily food and water intake and to avoid any leisure sport activity or self-administered exercise throughout the study period. Players were required to wear their usual training uniforms and football boots during the tests, performed in their respective habitual training venues.

**Table 1:** Schematic representation of a training week before the intervention period in young (YT) and professional (PT) soccer players.

	Monday	Tuesday	Wednesday	Thursday	Friday	Saturday	Sunday
YT	Strength/power and injury prevention		RSA test, small sided games (4 vs 4 to 6 vs 6), aerobic power and tactical drills		Yo-Yo test, speed/reaction soccer and tactical work game		Official match
PT	Strength/power and injury prevention		RSA test, small sided games (4 vs 4 to 6 vs 6), aerobic power and tactical drills	Strength/power and tactical work game (match simulation)	Yo-Yo test, speed/reaction soccer and strategy drills	Activation	Official match

RSA: repeated-sprint ability

### Participants

Male professional senior (n = 17, PT; age: 24.0 ± 2.8 years; height: 179.6 ± 1.8 cm; body mass: 74.5 ± 4.6 kg; VO_2_max: 58.29 ± 3.0 ml·kg^-1^·min^-1^) and young soccer players (n = 16, YT; age: 18.3 ± 0.8 years; height: 173.5 ± 9.9 cm; body mass: 65.4 ± 1.3 kg; VO_2_max: 54.65 ± 2.1 ml·kg^-1^·min^-1^) volunteered for the investigation. Players from both teams had a minimum soccer experience of 7 years at the commencement of the study. Bout groups performed three (YT) to four (PT) training sessions and one national-level match per week, in the three months preceding this study. Written informed consent was signed by all players (and their parents or guardians for under 18 years of age athletes) after a brief but detailed explanation about the aims, benefits, and risks involved with this investigation. The study was conducted according to the Declaration of Helsinki and the Institutional Research Ethics Committee (Universidad Isabel I) granted approval for the study.

### Yo-Yo intermittent recovery test

Athletes completed the YYIR1 test as previously described [26]. Running speed was set through an acoustic signal amplified by speakers (SONY-ENG203^®^, Japan), connected to a notebook (Acer TravelMater 5720^®^, Taiwan). Maximal heart rate was measured with a heart rate monitor (Polar Team Sport System^®^, Polar Electro Oy, Finland) and total distance was recorded by the number of runs. The test was finished when i) subjects were unable to complete two consecutive 20-m runs at the pace dictated by the acoustic signal, or ii) when athletes achieved volitional exhaustion [28]. Subjects were instructed and motivated to achieve maximal effort during testing (validated by the achievement of ±10% of predicted maximal heart rate).

### Repeated-sprint ability test

The RSA test involved eight maximal 30-m sprints, separated by 25 seconds of active recovery between sprints. Approximately two seconds before each sprint subjects assumed the start position [14] with the front foot placed 0.5 m behind the first photocell (DSD Laser System^®^, Leon, Spain), as previously described [23]. Immediately after the warm-up, each player completed a single *criterion-reference* sprint and this trial was used as the criterion score during the subsequent sprints [4]. Then, athletes rested for 5 minutes before commencement of the RSA test. If the first sprint-time of the RSA test was 2.5% greater (i.e., worse) than the *criterion-reference* sprint time, subjects were requested to rest for further 5 minutes and then restart the test. The RSA_best_, RSA_mean_, RSA_total_ and *Sdec* [4,5,14] were determined. The *Sdec* was calculated as (RSA_total_/(RSA_best_ × total number of sprints) × 100)– 100 [26].

### Statistical analyses

The results are expressed as mean ± standard deviation (*SD*). The change in tests performance is presented as percentage (Δ = [after value—before value] / before value). In addition to the comparison between PT and YT, the median split technique was used to divide the pooled participants into fast (FG) and slow (SG) performers, according to the median value calculated to RSA_best_ [5,27]. Normal distribution of data was confirmed by using Kolmogorov-Smirnov test and normal probability plot. A two-way ANOVA with repeated measures on time [before vs. after intervention] × team [professional vs. young players]) and time [before vs. after intervention] × group [fast vs. slow players] was used to analyse results. When a significant *F* value was found, Bonferroni’s post hoc test was applied to establish differences between means. Cohen’s d effect size (ES) was calculated and qualitatively assessed as trivial (0–0.19), small (0.20–0.49), medium (0.50–0.79) and large (0.80 and greater) [28]. The p<0.05 criterion was used to establish statistical significance. Analyses were performed using standard statistical software (SPSS, v.17.0, Chicago, Illinois, USA).

## Results

### Yo-Yo intermittent recovery test

The YYIR1 performance was not significantly affected (p > 0.05) by the in-season break (PT: before 2,368 ± 265 m, after 2,256 ± 283 m ES = 0.42; YT: before 2,054 ± 289 m, after 1,986 ± 321 m ES = 0.23).

### Repeated-sprint ability test

Regarding the effects of in-season break according to playing level, before detraining, PT showed significantly (p < 0.05) better RSA_best_ than YT (~ 2.8%) (Table 2). The RSA_best_, RSA_mean_ and RSA_total_ performance was worsened after the in-season break in both YT (p < 0.05) and PT (p < 0.01) (Table 2), with no difference between PT and YT.

**Table 2:** RSA in professional (PT; n = 17) and young elite (YT; n = 16) soccer players.

		Before	After	Δ	ES
RSA_best_ (s)	YT	4.03 ± 0.15	4.11 ± 0.14 *	1.9 ± 3.0	1.03 (large)
	PT	3.92 ± 0.11 †	4.04 ± 0.13 **	3.0 ± 2.7	1.03 (large)
RSA_mean_ (s)	YT	4.19 ± 0.12	4.26 ± 0.17 *	1.7 ± 2.6	0.65 (medium)
	PT	4.12 ± 0.12	4.22 ± 0.12 **	2.3 ± 2.6	1.03 (large)
RSA_total_ (s)	YT	33.52 ± 0.97	34.12 ± 1.40 *	1.7 ± 2.6	0.51 (medium)
	PT	32.91 ± 0.91	33.80 ± 0.94 **	2.3 ± 2.6	1.03 (large)
*Sdec* (%)	YT	3.90 ± 1.65	3.69 ± 1.61	-0.21 ± 2.2	0.13 (trivial)
	PT	5.21 ± 1.91	4.48 ± 2.14	-0.73 ± 2.4	0.36 (small)

RSA: repeated sprint ability; RSA_best_, RSA_mean_ and RSA_total_: best, mean and total time in the RSA test, respectively; *Sdec*: percentage decrement score; Δ: percentage change; ES: effect size.

*, **: denote difference compared with before values (p < 0.05 and p < 0.01, respectively);

^†^: denote difference between teams (p < 0.05).

Regarding the effects of in-season break according to baseline RSA performance, the median of the RSA_best_ was 3.95 s (27.3 km·h^-1^), categorizing players into FG (< 3.95 s) and SG (≥ 3.95 s) (Table 3). The FG group (n = 14) was composed of 8 professional and 6 young soccer players. The SG (n = 19) was composed of 9 professional and 10 young soccer players. Before detraining, the FG showed better performances in RSA_best_ (~ 6.8%), RSA_mean_ (~ 4.1%), and RSA_total_ (~ 4.1%), (p < 0.01; Table 3), confirming the appropriateness of the median split technique. The players from the FG showed a greater impairment in the RSA_best_ (ES = 2.09), RSA_mean_ (ES = 1.04) and RSA_total_ (ES = 1.46) than the players from the SG (ES = 0.56, 1.02, 0.58, respectively) (Table 3).

**Table 3:** RSA in slow (SG, n = 19) and fast (FG, n = 14) performers^€^.

		Before	After	Δ	ES
RSA_best_ (s)	SG	4.08 ± 0.02	4.14 ± 0.03 *	1.5 ± 2.6	0.56 (medium)
	FG	3.82 ± 0.02 ††	3.98 ± 0.04 **	4.0 ± 2.5 †	2.09 (large)
RSA_mean_ (s)	SG	4.23 ± 0.02	4.31 ± 0.03 *	2.0 ± 2.6	1.02 (large)
	FG	4.06 ± 0.03 ††	4.16 ± 0.04 **	2.2 ± 2.5	1.04 (large)
RSA_total_ (s)	SG	33.81 ± 0.19	34.49 ± 0.26 *	2.0 ± 2.6	0.58 (medium)
	FG	32.45 ± 0.7 ††	33.21 ± 0.29 **	2.2 ± 2.5	1.46 (large)
*Sdec* (%)	SG	3.64 ± 0.52	4.25 ± 2.01	0.5 ± 1.9	0.29 (small)
	FG	5.91 ± 1.92 ††	3.93 ± 1.84 *	-2.0 ± 2.2 †	1.13 (large)

^€^: the median split technique was used to divide subjects into FG and SG performers, according to median RSA_best_ of 3.95 s; RSA: repeated sprint ability; RSA_best_, RSA_mean_ and RSA_total_: best, mean and total time in the RSA test, respectively; *Sdec*: percentage decrement score; Δ: percentage change; ES: effect size.

*, **: denote difference compared with before values (p < 0.05 and p < 0.01, respectively);

^†, ††^: denote difference between SG and FG (p < 0.05 and p < 0.01, respectively).

## Discussion

The main aim of the study was to analyse if initial performance level (sprinting speed) of soccer players can modulate repeated-sprint ability (RSA) and intermittent endurance changes during 2-weeks of detraining (i.e., in-season break). The major findings showed detrimental changes in RSA, but not intermittent endurance performance, in both PT and YT. Also, RSA_best_ was greatly impaired in FG compared to SG players (p<0.05, with a large 2.09 ES versus medium 0.56 ES), as well as RSA_total_ (large 1.46 ES versus medium 0.58 ES).

To our knowledge, this is the first controlled study showing the response of YYIR1 and RSA performance after an in-season break. However, a previous study [29] analysed the effects of a 2-weeks in-season break on male professional (age, 24.3 ± 4.2 years) Australian Football player’s fitness, including skinfolds, and heart rate plus rating of perceived exertion while plyers performed submaximal running velocity (12 km·h^-1^), high-intensity intermittent running exercise, and a standardized handball game. The aforementioned study showed increased levels of strength and cardiorespiratory fitness, despite a small increase in skinfold thickness. However, the athletes did not fully stop training, contrary to our study where soccer players interrupted training completely. In addition, in the aforementioned study, the authors did not control the training that athletes completed during the break. It is possible that the break allowed players to come back in January well recovered with preserved or even increased levels of strength and cardiorespiratory fitness.

According to our results, a complete reduction of training during 2-weeks of in-season break did not affect YYIR1 performance in PG or YG. Accordingly, high-intensity intermittent endurance performance might be more resilient to detraining [30] than some of its physiological correlates, such as maximal oxygen uptake (VO_2_max) [31], and other factors affecting high-intensity intermittent endurance performance [13].

Of note, FG players showed greater impairment in RSA_best_ performance after detraining compared to SG players. Due to their greater initial performance level, FG players may have experienced greater detraining effects [31], with increased negative effects on fast-twitch muscle fibers [32], the ability to use ATP and phosphocreatine [33], accompanied with greater production of metabolic by-products [34], which may negatively affect motor units recruitment and synchronization [35], and thus RSA [36]. In a similar study, faster futsal players assessed at the beginning of the pre-season also lost more of their sprinting speed than their slower peers [37]. In summary, independent from age, compared to slower players, faster players seem to be more negatively affected in their RSA by detraining. Therefore, fast team sports players may need more attention from technical staffs due to their tendency to lose their sprint quality in several phases of the preparation.

The FG players showed an enhanced *Sdec* performance after detraining. Although tapering effects may help explain this result [38], no other RSA value was improved after detraining. Previous studies have found worsening (5.8 ± 2.8% vs 7.8 ± 3.2%, p = 0.04) or maintenance (5.9 ± 2.3% vs 7.6 ± 2.8%) of *Sdec* after one [11] or two [12] weeks of detraining, respectively. Rapid RSA impairment with detraining might be related to reductions in resting phosphorylation status of the Na^+^-K^+^ pump [18]. The different RSA testing protocols used between studies may partially help to explain the relatively different results. However, the relationship between *Sdec* and the performance achieved in the first sprint may also contribute to the different results. In this sense, a slower first sprint (i.e., impaired sprint performance after detraining) will induce a reduction in *Sdec* values[39], which is translated into a *false-positive* improvement of *Sdec*. Thus, a more probable explanation stems on the poor validity and sensitivity of *Sdec* to detect actual performance impairment during RSA test and negative changes expected in response to short-term detraining. Although *Sdec* have been presented as a reliable marker of RSA performance [26], its use have been questioned, given that *Sdec* is the least reliable parameter calculated from RSA tests [4]. In fact, *Sdec* have showed poor sensitivity to training [3]. Hence, it appears that *Sdec* is a poor indicator of RSA performance changes in response to an in-season break period in soccer. It is possible that, for a better assessment of *Sdec*, the ideal sprint performance at the moment of RSA measurement should not be considered, but to take into account the better sprint of the athlete for the given sprint distance.

One potential limitation of this study was the estimation of VO_2_max through the YYIR1 test, since it could not be determined during laboratory-based maximal graded test. The direct measurement of VO_2_max (and metabolic thresholds) could offer more physiological information regarding the effects of detraining in soccer.

## Conclusion

A 2-week in-season break (detraining) period impaired RSA, with no effect on intermittent endurance in professional and elite young soccer players, with greater impairment of RSA_total_ and RSA_best_ in faster players, independent of their age category. In addition, *Sdec* does not seem to be sensitive to changes in RSA after a 2-week in-season break. Coaches should take these findings into consideration for appropriate training schedule after in-season break in order to regain the performance indices lost during the break.

## Practical applications

According to our results (poorer RSA after a 2-weeks in-season break), coaches and practitioners should considered an individualization of training loads during such periods, considering principles such as the minimal-effective dose, especially for players with greater fitness level, as these may be negatively affected to a greater magnitude by short-detraining periods.

## Supporting information

S1 File DatosGlobal. (XLSX)

S2 File Datos. (SAV)

S1 Dataset DataSet. (XLSX)
